# Absent right iliac bone on Tc99m MDP bone scan in a patient with Gorham's vanishing bone disease

**DOI:** 10.4103/0972-3919.63596

**Published:** 2010

**Authors:** Saleh Othman

**Affiliations:** Department of Radiology and Medical Imaging, King Khalid University Hospital and College of Medicine, King Saud University, Riyadh, Saudi Arabia

**Keywords:** Bone scan, Gorham's disease, iliac bone

## Abstract

Gorham's (vanishing bone) disease is an extremely rare condition of the bone. The diagnosis is usually made on the basis of the characteristic history of osteolysis and failure of bone healing in conjunction with the histological findings of marrow fibrosis and increased vascularity. When the disease is established, an X-ray and magnetic resonance imaging show complete loss of affected bone. There are very few reports found in literature on bone scan appearance of the disease. A bone scan of a 24-year-old female patient with known Gorham's disease revealed absence of tracer uptake in the right iliac bone, right sacroiliac joint, and part of the right ischial pubic rami, which matched the radiographic abnormalities. Consequently this disease should be added to the gamut of cold defects seen on bone scan.

## INTRODUCTION

Gorham's (vanishing bone) disease is an extremely rare condition of the bone. The diagnosis is usually made on the basis of the characteristic history of osteolysis and failure of bone healing in conjunction with the histological findings of marrow fibrosis and increased vascularity. When the disease is established an X-ray and MRI show complete loss of affected bone. Very few reports are found in literature on bone scan appearance of the disease.

## CASE REPORT

A bone scan was performed on a 24-year-old female patient with known Gorham's disease. This was correlated with a pelvic radiograph and corresponding MRI.

**Figure 1:**
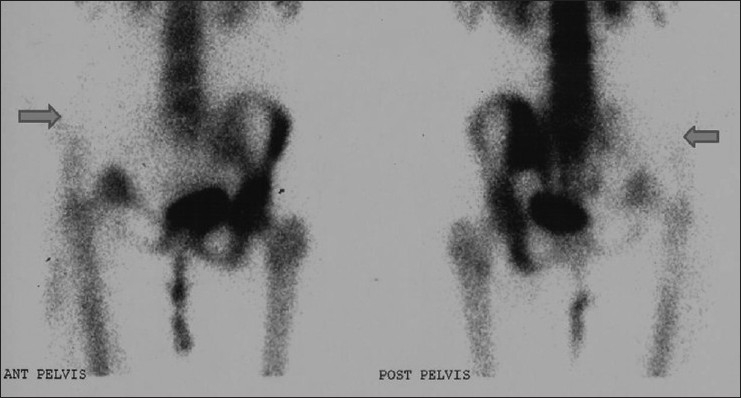
Tc99m MDP bone scan (anterior and posterior pelvic views) showing absence of tracer uptake in the right iliac bone, right sacroiliac joint, and part of the right ischial pubic rami (arrows)

**Figure 2:**
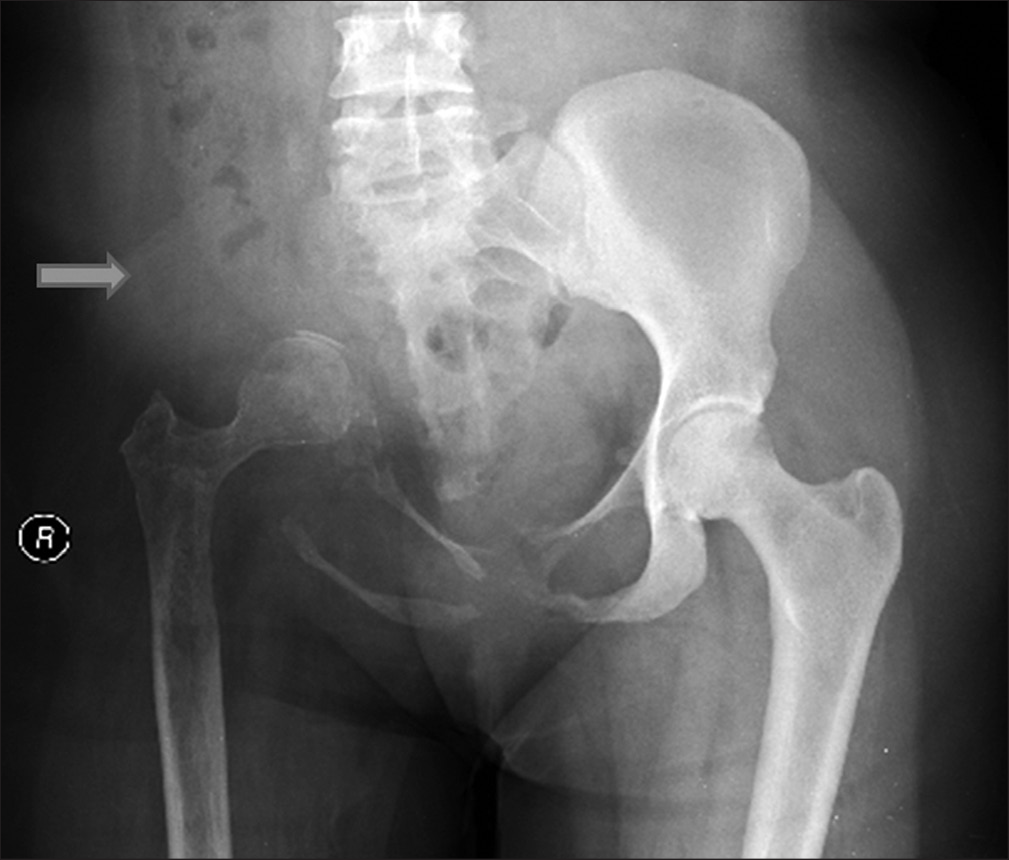
Pelvic X-ray shows absent bone tissue in the right iliac bone, right sacroiliac joint, and part of the right ischial pubic rami (arrow).

Images showed absence of tracer uptake in the right iliac bone, right sacroiliac joint, and part of the right ischial pubic rami, indicating absence of bone in the mentioned regions, which matched the absent bone tissue on the pelvic X-ray [Figure 1 and 2].

## DISCUSSION

Cold defects on bone scan have been reported in many disease conditions.[1] Gorham's disease should be included in this list. Gorham's disease, which has many other synonyms (Disappearing bone disease, Gorham's syndrome, Gorham-Stout syndrome, Vanishing bone disease, Phantom bone disease, Massive osteolysis, and others) is an extremely rare condition of the bone, with less than 200 cases in literature since the condition was originally reported. Few of the reported cases describe ragiological and scintigraphic findings in this disease condition.[2‐11] Consequently we consider that Gorham's disease should be added to the gamut of cold defects seen on bone scan.

## References

[1] Datz F (1995). Cold defects in bone scanning. Gamut in Nuclear medicine.

[2] Dan'ura T, Ozaki T, Sugihara S, Taguchi K, Inoue H (1998). Massive osteolysis in the pelvis: A case report. Acta Orthop Scand.

[3] Rauh G, Gross M (1997). Disappearing bone disease (Gorham-stout disease): Report of a case with a follow-up of 48 years. Eur J Med Res.

[4] Nemec B, Matovinovic D, Gulan G, Kozic S, Schnurrer T (1996). Idiopathic osteolysis of the acetabulum: A case report. J Bone Joint Surg Br.

[5] Stove J, Reichelt A (1995). Massive osteolysis of the pelvis, femur and sacral bone with a Gorham-Stout syndrome. Arch Orthop Trauma Surg.

[6] Kulenkampff HA, Richter GM, Hasse WE, Adler CP (1990). Massive pelvic osteolysis in the Gorham-Stout Syndrome. Int Orthop.

[7] Spieth ME, Greenspan A, Forrester DM, Ansari AN, Kimura RL, Gleason-Jordan I (1997). Gorham's disease of the radius: Radiographic, scintigraphic and MRI findings with pathologic correlation: A case report and review of the literature. Skeletal Radiol.

[8] Igel BJ, Shah H, Williamson MR, Sell JJ (1994). Gorham's syndrome: Correlative imaging using nuclear medicine, plain film, and 3-D CT. Clin Nucl Med.

[9] Kobayashi H, Shigeno C, Sakahara H, Hosono M, Hosono M, Yao ZS (1994). Intraosseous hemangiomatosis: Technetium-99m(v)dimercaptosuccinic acid and technetium-99m-hydroxymethylene diphosphonate imaging. J Nucl Med.

[10] Dominguez R, Washowich TL (1994). Gorham's disease or vanishing bone disease: Plain film, CT, and MRI findings of two cases. Pediatr Radiol.

[11] Marymont JV (1987). Comparative imaging: Massive osteolysis (Gorham's syndrome, disappearing bone disease). Clin Nucl Med.

